# Prognostic evaluation of non-muscle invasive bladder cancer with P-CRP and its nomogram

**DOI:** 10.3389/fonc.2025.1406585

**Published:** 2025-02-03

**Authors:** Junyun Wu, Zhixuan Deng, Xu Lei, Zhiyao Xu, Chenxi Tan, Yunqiao Tang, Xi Sheng, Ning Yang

**Affiliations:** ^1^ The Second Affiliated Hospital of University of South China, Hengyang, Hunan, China; ^2^ Institute of Cell Biology, Hengyang Medical School, University of South China, Hengyang, Hunan, China; ^3^ The Central Hospital of Shaoyang, Shaoyang, Hunan, China; ^4^ Hengyang Medical School, University of South China, Hengyang, Hunan, China

**Keywords:** non-muscle invasive bladder cancer, P-CRP, prognostic analysis, nomogram, recurrence-free survival

## Abstract

**Purpose:**

To investigate the impact of the product of preoperative platelet count and C-reactive protein (P-CRP) on the postoperative prognosis of patients with non-muscle invasive bladder cancer (NMIBC), and to construct a Nomogram to predict the recurrence-free survival (RFS) of NMIBC patients based on pathological data.

**Methods:**

A retrospective analysis was conducted on the clinical data of 164 NMIBC patients who underwent transurethral resection of bladder tumors (TURBT) at the Second Affiliated Hospital of University of South China from January 2013 to December 2019. The endpoint of the study was the RFS. Kaplan-Meier (KM) method and Cox regression were used for analysis to identify independent factors affecting RFS. Then, the Nomogram was used to visualize the results of the multivariate analysis that were statistically significant and related to the RFS of NMIBC patients. Finally, the predictive ability of the model was evaluated using the concordance index (C-index) and calibration curves.

**Results:**

Before the end of the follow-up, the RFS was 88.3% at 1 year, 75.5% at 2 years, and 58.5% at 3 years. KM curves showed that P-CRP (HR=0.357, 95% CI: 0.204-0.625, P<0.001), number of tumors (HR=2.658, 95% CI: 1.572-4.494, P<0.001), tumor size (HR=2.271, 95% CI: 1.377-3.745, P=0.001), T stage of the tumor (HR=2.026, 95% CI: 1.233-3.329, P=0.005), and tumor G grade (G2: HR=1.615, 95% CI: 0.48-5.433, G3: HR=3.361, 95% CI: 1.022-11.054) were independent factors affecting the RFS of NMIBC patients after TURBT. The Nomogram could estimate the risk of tumor recurrence at 1, 2, and 3 years postoperatively. The Nomogram model incorporating P-CRP parameters had a higher predictive accuracy than the classic model that only included EORTC risk group parameters.

**Conclusion:**

Preoperative P-CRP has a certain impact on the RFS of NMIBC patients after TURBT. The Nomogram incorporating P-CRP, number of tumors, tumor size, T stage, and tumor pathological grading can better predict the postoperative recurrence risk of NMIBC patients.

## Introduction

Bladder cancer (BC) ranks 12th in incidence among all tumors, and its incidence in male malignant tumors ranks 7th, with mortality ranking 9th (1, 2). In female patients, the incidence and mortality are approximately one-fourth of those in male patients. BC can be divided into two major categories: non-muscle invasive bladder cancer (NMIBC) and muscle invasive bladder cancer (MIBC). The primary treatment option for NMIBC is surgery combined with postoperative bladder instillation therapy. However, the probability of recurrence remains high after treatment (3–5). The most commonly used prognostic prediction model for NMIBC is the risk assessment system introduced by the European Organization for Research and Treatment of Cancer (EORTC) (6). However, this system’s scoring criteria are mainly based on pathological data, and its predictive ability is poor when the clinical stage of the tumor is not determined. Therefore, we hope to construct a more accurate and convenient prognostic prediction model for NMIBC patients.

The product of platelet count and C-reactive protein (Platelet*C-reactive Protein, P-CRP) is calculated as the peripheral platelet count (/ul) multiplied by the serum CRP level (mg/dL) divided by 10^4. Studies have shown that in patients with rectal cancer, gastric cancer, and pancreatic cancer, the P-CRP value is a known synergistic prognostic indicator, which is more important than CRP or platelet levels alone (7–9).

The Nomogram is a visual mathematical model (10). In medical oncology research, the Nomogram can incorporate factors such as the patient’s general condition, examination results, and surgical situation, and calculate the patient’s prognosis through a formula. The recurrence probability of NMIBC is relatively high, so many scholars use RFS as the endpoint of their research (11–13). However, there is currently little research on the correlation between P-CRP and RFS in NMIBC. This study aims to clarify the impact of preoperative P-CRP levels in NMIBC patients on postoperative RFS and to construct a Nomogram model that can individualize the prediction of RFS for NMIBC patients after TURBT, incorporating relevant factors from the EORTC risk group.

## Methods

### Inclusion and exclusion criteria

We conducted a retrospective analysis of 164 cases of NMIBC patients who underwent TURBT at the Department of Urology, the Second Affiliated Hospital of University of South China, from January 1, 2013, to December 31, 2019. Inclusion criteria were: (1) preoperative complete blood examination reports; (2) complete clinical follow-up data; (3) all patients had primary NMIBC with the pathological staging of Ta or T1; (4) patients with cM0 disease; and (5) postoperative regular bladder instillation according to guideline standards. Exclusion criteria were: (1) concurrent non-tumor diseases, such as immune diseases; (2) concurrent other malignant tumors; (3) previous surgical treatment for bladder lesions; (4) other types of bladder diseases, such as neurogenic bladder; (5) pathological diagnosis of non-urothelial cell carcinoma; (6) preoperative infection; (7) concurrent inflammatory chronic disease; (8) concurrent carcinoma *in situ* (CIS), and (9) severe cognitive impairment. Strictly following our inclusion and exclusion criteria, we ultimately selected the data of 162 patients.

### Data collection

We retrospectively collected the clinical and pathological data of 164 NMIBC patients. Each enrolled patient had to have a complete cystoscopy, ultrasound, CT scan results, and routine blood tests (including complete blood count, urinalysis, and biochemical tests) before surgery. This study determined the pathological G grading of NMIBC based on the 1973 World Health Organization guidelines and the TNM staging of tumors based on the 2017 American Cancer Society report. By reviewing the electronic medical record system of the Second Affiliated Hospital of University of South China, we obtained all clinical data of the patients, including age, gender, history of hypertension, results of blood tests, pathological tumor staging, etc., as well as disease information obtained through follow-up. The tumor size was defined as the maximum diameter of the specimen after TURBT, and P-CRP was calculated as the peripheral platelet count (/ul) multiplied by the serum CRP level (mg/dL) divided by 10^^^4. According to previous literature and the consensus of Chinese experts on intravesical therapy for non-muscle invasive bladder cancer, all patients with pT1 stage and G3 stage bladder cancer underwent repeat transurethral resection of the bladder (reTURB) (14, 15). All patients were required to have regular follow-up examinations postoperatively, including complete blood count, urinalysis, blood biochemical tests, cystoscopy, etc. The follow-up protocol was to conduct a check-up every 3 months for the first two years after surgery and every 6 months thereafter. The postoperative bladder instillation regimen should be determined according to guideline standards: (1) The low-risk group received a single dose of intravesical chemotherapy immediately after surgery, without subsequent bladder induction or maintenance instillations. (2) The intermediate-risk group received a single dose of intravesical chemotherapy immediately after surgery, followed by bladder induction and maintenance instillations, with the total duration not exceeding one year. (3) For patients in the high-risk group, Bacillus Calmette-Guérin (BCG) intravesical instillation is recommended. BCG instillation begins 2 weeks after surgery, administered at a dose of 120 mg per instillation. After 6 weekly sessions of induction perfusion, enhanced perfusion is performed once every 2 weeks for a total of 3 times. Maintenance perfusion is then initiated once a month for a total of 10 times. The entire instillation regimen lasts 1 year, totaling 19 instillations. If the patient refuses BCG, intravesical chemotherapy is initiated immediately after surgery, followed by bladder induction and maintenance instillations, with a total duration not exceeding 1 year (15). The drug used for intravesical chemotherapy is pirarubicin, administered at a dose of 30-50 mg per instillation. In cases where BCG therapy failed, patients were categorized into BCG-refractory, BCG-resistant, BCG-relapsing, or BCG-intolerant groups. The treatment for BCG failure included: (1) BCG-refractory: Patients who do not respond to an initial induction course and subsequent maintenance or repeat induction courses are considered for radical cystectomy. Alternative intravesical therapies, such as pirarubicin or mitomycin, can also be considered for patients who are not surgical candidates. (2) BCG-resistant: Patients who initially respond to BCG but later develop recurrence are treated with additional courses of BCG. If the disease recurs after a second BCG course, these patients might then be managed similarly to BCG-refractory cases, with consideration for radical cystectomy. (3) BCG-relapsing: It applies to patients who relapse after achieving an initial complete response to BCG therapy. Early relapses (within 6 months) are managed more aggressively and will be with cystectomy, while late relapses will be treated with repeat BCG therapy.

### Study endpoint and EORTC risk scoring system

In this study, Recurrence-Free Survival (RFS) served as the primary endpoint. RFS is defined as high-grade (HG) recurrence or progression to pT2 or radical cystectomy. Currently, the most convenient and relatively accurate prognostic model for NMIBC patients is the EORTC risk scoring system proposed by European scholars, which includes the following content: (1) low-risk group: Must meet all the following conditions simultaneously: primary, single, Ta, low grade or G1 stage, tumor diameter <3cm, and no associated CIS (carcinoma *in situ*); (2) intermediate-risk group: Patients who are not included in either the high-risk or low-risk groups; (3) high-risk group: Meets any one of the following conditions: a: T1 stage; b: high grade/G3 stage; c: CIS; d: meets all of the following simultaneously: multiple, recurrent, diameter >3cm, and TaG1/G2 stage.

### Statistical methods

This study utilized SPSS 25.0 and R software version 4.1.1 for data analysis and model construction. Continuous variable-type clinical data were statistically analyzed using the t-test, and categorical variable-type clinical data were examined using the chi-square test. Additionally, we determined the optimal cutoff value for P-CRP by identifying the point in the upper left corner of the ROC curve (i.e., the point with the maximum Youden’s index). In subsequent survival analyses, we used the Kaplan-Meier (KM) curve to compare the RFS between different groups and employed the log-rank test to verify the conclusions. Furthermore, based on the Cox regression model, we constructed a Nomogram that can predict the RFS at 1, 2, and 3 years, thereby linking various factors affecting the prognosis of NMIBC patients with RFS. This allows us to predict the RFS after TURBT based on the patients’ clinical indicators. Moreover, we can visually assess the predictive ability of our Nomogram through calibration curves and quantitatively evaluate the predictive power of the Nomogram using the concordance index (C-index). To further evaluate and compare our prognostic tool with other recognized inflammatory response markers, we used ROC curves. The inflammatory response markers included Neutrophil-to-Lymphocyte Ratio (NLR), Platelet-to-Lymphocyte Ratio (PLR), Systemic Immune-Inflammation Index (SII), Prognostic Nutritional Index (PNI), Albumin-to-Globulin Ratio (AGR), and P-CRP. By comparing the Area Under the Curve (AUC) values of these ROC curves, we assessed the predictive accuracy of our tool relative to these established markers. Additionally, we tested the relationship between prognosis and other covariates, including drinking habits, alcohol consumption history, and other patient demographics. These covariates were included in univariate and multivariate analyses to determine their impact on Recurrence-Free Survival (RFS). The univariate analysis was performed using the Kaplan-Meier method and the log-rank test to compare differences in RFS rates across different covariates. The multivariate analysis was conducted using the Cox regression model to identify independent risk factors influencing RFS. In this study, all statistical analyses were performed using two-sided tests, and a P-value of less than 0.05 was considered statistically significant.

## Results

### Patient demographics and pathological features

We collected data on 164 patients with NMIBC who underwent TURBT and met our study inclusion criteria from January 1, 2013, to December 31, 2019 (**Table 1**).

**Table 1:** General data and pathological features of patients with NMIBC.

All patients (N = 164)	
Parameters	N (%)
Age (years)	
≥65	86(52.4)
<65	78(47.6)
Sex	
Male	131(79.9)
Female	33(20.1)
Hypertension history	
Exist	38(23.2)
N/A	126(76.8)
Smoking history	
Exist	65(39.6)
N/A	99(60.4)
Tumor size (cm)	
≤3 cm	119(72.6)
>3 cm	45(27.4)
Number	
Single	127(77.4)
Multiple	37(22.6)
T stage	
Ta	77(47.0)
T1	87(53.0)
G grade	
G1	20(12.2)
G2	74(45.1)
G3	70(42.7)
Type of maintenance instillation	
Pirabucin	148(90.2)
BCG	16(9.8)

### Value of P-CRP in predicting RFS

**Figure 1:**
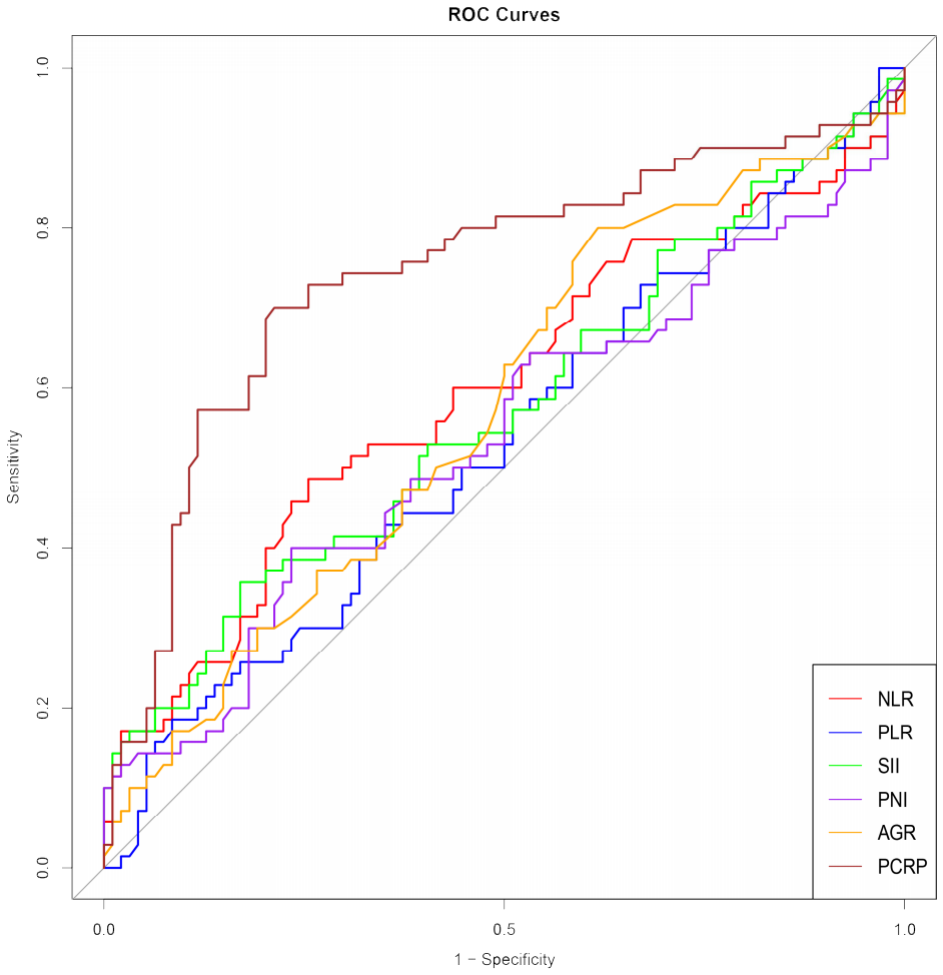
ROC curves of inflammatory response markers NLR, PLR, SII, PNI, AGR, P-CRP. NLR, Neutrophil-to-Lymphocyte Ratio; PLR, Platelet-to-Lymphocyte Ratio; SII, Systemic Immune-Inflammation Index; PNI, Prognostic Nutritional Index; AGR, Albumin-to-Globulin Ratio.

The average preoperative platelet count ± standard deviation of the enrolled patients was 192.5 ± 59.9 * 10^9 per L, and the average preoperative CRP ± standard deviation was 5.9 ± 5.5 mg/L. We calculated the values of individual patients’ inflammatory response markers using the formula and plotted the ROC curves (including P-CRP and other related inflammatory markers) (**Figure 1**) (**Table 2**). We found that among all inflammatory markers, P-CRP had the highest AUC value. Using the ROC working curve, we identified the point corresponding to the maximum Youden’s index, which was associated with a P-CRP value of 8.36. Patients were divided into two groups based on this cutoff value. There were 75 patients with P-CRP ≥ 8.36, accounting for 45.7% of the total, defined as the high P-CRP group; and 89 patients with P-CRP < 8.36, accounting for 54.3%, defined as the low P-CRP group.

**Table 2:** AUC area under ROC curve of inflammatory response markers NLR. PLR. Sil, PNI, AGR and P-CRP.

Test the result of the variable	AUC	Standard error	Asymptotic significance	Asymptotically 95%	Confidence interval	Optimal cut-off value
	area	a	b	lower limit	upper limit	
NLR	0.594	0.046	0.039	0.504	0.685	3.960
PLR	0.469	0.046	0.492	0.378	0.559	224.200
Sil	0.570	0.046	0.127	0.479	0.661	920.810
PNI	0.464	0.047	0.435	0.372	0.557	251.700
AGR	0.426	0.045	0.100	0.337	0.515	3.030
P-CRP	0.743	0.042	0.000	0.661	0.824	8.360

### Comparison of general information between different P-CRP level groups in NMIBC

A comparison of general information between different P-CRP level groups in NMIBC revealed that there were no statistically significant differences in terms of age, gender, smoking history, alcohol consumption history, hypertension history, and diabetes history (P > 0.05).

### Comparison of biochemical tests between different P-CRP level groups in NMIBC

A comparison of biochemical test data between different P-CRP level groups in NMIBC showed significant differences in hemoglobin, red blood cell count, white blood cell count, and CRP levels among the various P-CRP groups (P < 0.05). However, there were no statistically significant differences in liver and kidney function tests and lipid-related indicators between the two groups (P > 0.05) (**Table 3**).

**Table 3:** Comparison of blood biochemical levels among different P-CRP levels in NMIBC patients.

	High P-CRP group		Low P-CRP group		t value	P value
	Mean	SD	Mean	SD		
Blood biochemical parameters						
TP (g/L)	64.20	7.77	61.84	9.43	-1.758	0.081
DBIL (mg/L)	6.08	12.63	4.07	3.24	-1.341	0.184
IBIL (mg/L)	9.60	4.75	8.38	4.15	-1.738	0.084
ALT (U/L)	29.13	58.04	18.92	10.45	-1.629	0.105
AST (U/L)	31.01	35.93	22.45	6.87	2.201	0.290
UREA (mmol/L)	6.23	5.10	5.68	2.83	1.076	0.301
CREA (umol/L)	100.98	85.11	91.87	40.83	2.389	0.124
UA (umol/L)	344.21	99.69	333.25	101.47	0	0.999
TG (mmol/L)	1.72	1.47	1.78	1.40	0.397	0.53
CHOL (mmol/L)	4.28	1.07	5.47	7.67	1.129	0.29
HDL-C (mmol/L)	1.31	0.32	1.31	0.34	0.007	0.932
LDL-C (mmol/L)	2.71	0.82	2.72	0.74	0.184	0.669
LDH (U/L)	195.21	34.59	203.95	52.83	3.131	0.081
GLU (mmol/L)	6.08	12.63	4.07	3.24	0.299	0.58
ALP (U/L)	65.87	21.71	84.43	62.29	3.589	0.06

### Comparison of pathological data between different P-CRP level groups in NMIBC

**Figure 2:**
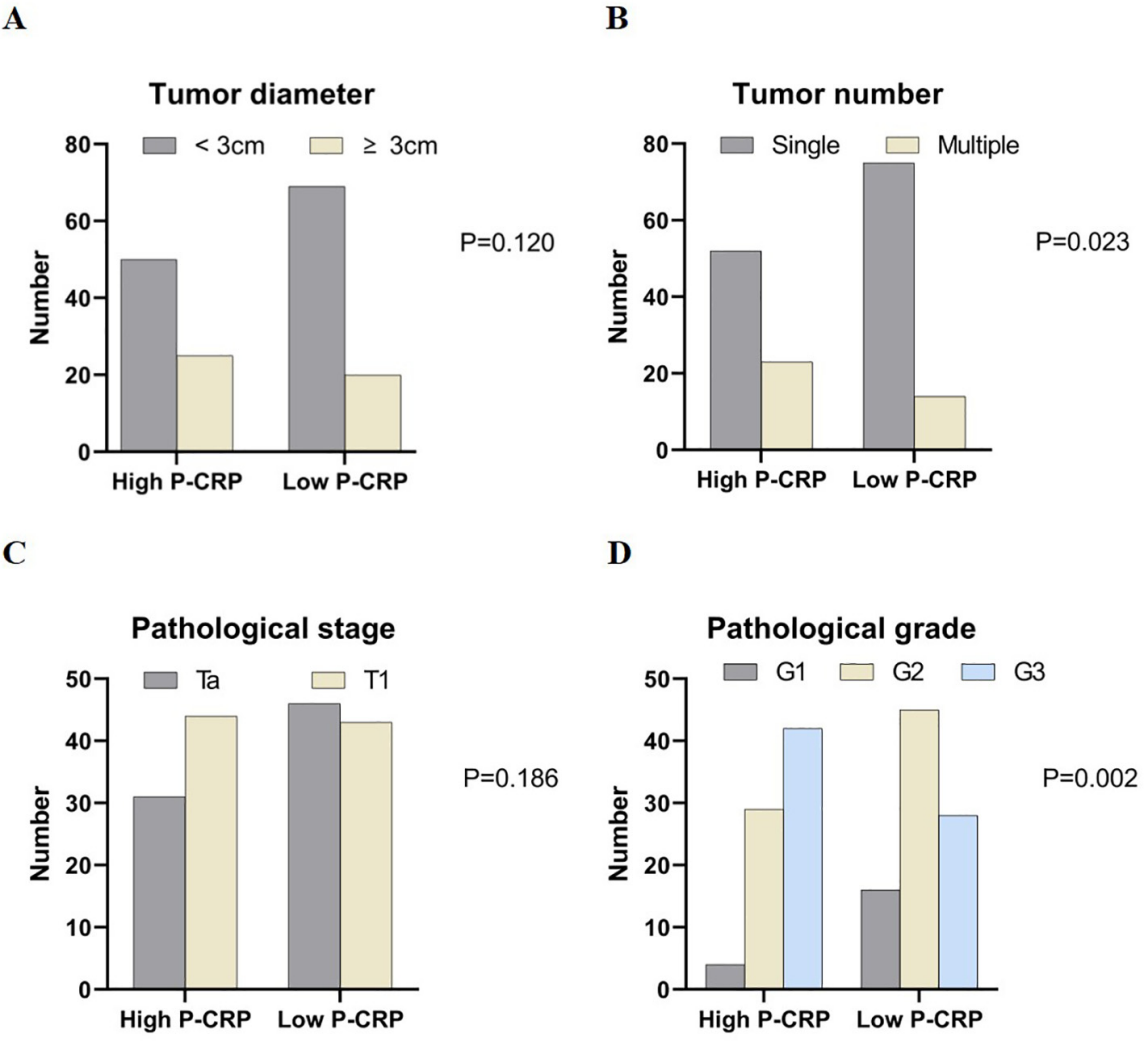
Comparison of case data between groups with different P-CRP levels in NMIBC patients. **(A)** Distribution of tumor diameter by P-CRP levels. **(B)** Distribution of tumor number by P-CRP levels. **(C)** Distribution of pathological stage by P-CRP levels. **(D)** Distribution of pathological grade by P-CRP levels.

When comparing the pathological data between different P-CRP level groups in NMIBC, it was found that there were significant differences in the number of tumors (single, multiple) and tumor pathological grading (G1, G2, G3) (P < 0.05). However, there were no significant differences in tumor diameter (<3cm, ≥3cm) and T stage (Ta, T1) between the different P-CRP level groups (P > 0.05) (**Figure 2**).

### Comparison of recurrence-free survival times between different P-CRP level groups

**Figure 3:**
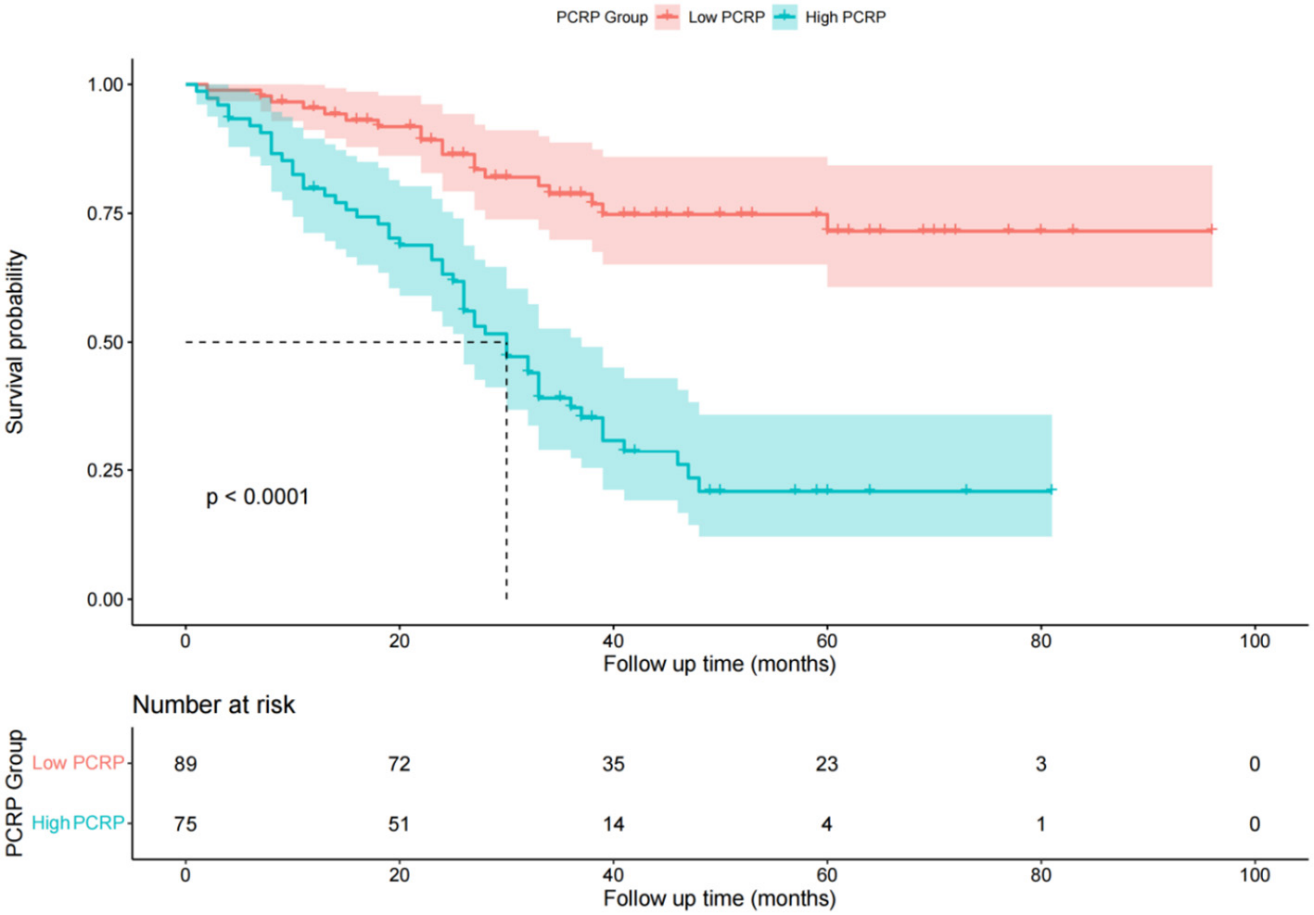
Survival function diagram of different P-CRP level groups.

In the low P-CRP group, the RFS rates at 1, 2, and 3 years were 95.4%, 86.4%, and 78.7%, respectively. The cumulative RFS did not fall below 50% during the follow-up period, with an average RFS of 77 months and a standard deviation of 4 months. In the high P-CRP group, the RFS rates at 1, 2, and 3 years were 85.2%, 63.2%, and 56.0%, respectively. The median RFS was 30 months, and the average RFS was 36 months with a standard deviation of 3 months (**Figure 3**). This suggests that the prognosis of the low P-CRP group is better than that of the high P-CRP group (P < 0.05).

### Comparison of general information among different EORTC risk groups

According to the EORTC scoring criteria, among the 164 patients with NMIBC, 8 were classified as low-risk group (4.8%), 25 as intermediate-risk group (15.2%), and 131 as high-risk group (80.0%). A comparison of general information between different EORTC risk groups revealed that there was a statistically significant difference in age among the groups (P < 0.05). However, differences in other general information, such as gender, smoking history, alcohol consumption history, hypertension history, and diabetes history, were not statistically significant across the groups (P > 0.05).

### Comparison of biochemical tests between different EORTC risk groups

Among the different EORTC risk groups, there were statistically significant differences in red blood cell count, hemoglobin, and triglyceride levels (P < 0.05) (**Table 4**).

**Table 4:** Comparison of blood biochemical tests among different risk groups of EORTC.

	Low-risk group		Medium-risk group		High-risk group		F	P
	Mean	SD	Mean	SD	Mean	SD		
Blood biochemical parameters								
WBC (×10°/L)	5.80	1.56	6.60	1.69	6.73	2.94	0.425	0.654
PLT (×10°/L)	194.75	59.11	204.76	54.93	190.02	61.07	0.634	0.530
NEU (×10°/L)	3.89	1.46	4.17	1.69	4.59	2.67	0.529	0.590
LY (×10°/L)	1.39	0.58	1.49	0.56	1.46	0.78	0.058	0.944
MONO (×10^9^L)	0.48	0.16	0.49	0.16	0.53	0.29	0.384	0.682
ALB (g/L)	41.33	5.20	40.85	4.15	39.00	6.80	1.251	0.289
GLOB (g/L)	59.90	14.09	65.71	10.27	62.57	7.99	1.867	0.158
CRP (mg/L)	23.59	4.59	26.34	5.69	24.48	6.58	1.027	0.360
TP (g/L)	5.86	6.29	4.26	3.99	5.08	9.69	0.127	0.880
DBIL (mg/L)	7.89	5.37	9.80	4.29	8.39	4.45	0.719	0.489
IBIL (mg/L)	20.78	13.15	19.49	8.15	24.50	44.58	0.183	0.833
ALT (U/L)	21.24	6.99	22.47	6.48	26.37	25.09	0.581	0.560
AST (U/L)	6.85	3.05	5.26	3.246	6.00	4.21	0.569	0.567
UREA (mmol/L)	120.46	87.90	81.19	22.35	97.37	68.47	1.253	0.288
CREA (umol/L)	391.25	83.41	361.92	91.69	330.50	101.96	2.228	0.111
UA (umol/L)	5.04	0.68	4.57	0.81	4.98	6.41	0.043	0.958
CHOL (mmol/L)	1.43	0.27	1.16	0.21	1.32	0.34	2.509	0.085
HDL-C (mmol/L)	3.14	0.70	2.90	0.64	2.65	0.79	2.016	0.138
LDL-C (mmol/L)	2.34	0.95	5.00	5.68	6.27	5.55	2.338	0.1
LDH (U/L)	207.33	35.01	189.38	46.37	201.98	46.06	0.443	0.644
GLU (mmol/L)	5.65	0.91	6.08	1.96	6.58	3.13	0.575	0.564
ALP (U/L)	67.83	25.75	63.75	17.97	76.23	49.19	0.579	0.562

### Comparison of recurrence-free survival times among different EORTC risk groups

**Figure 4:**
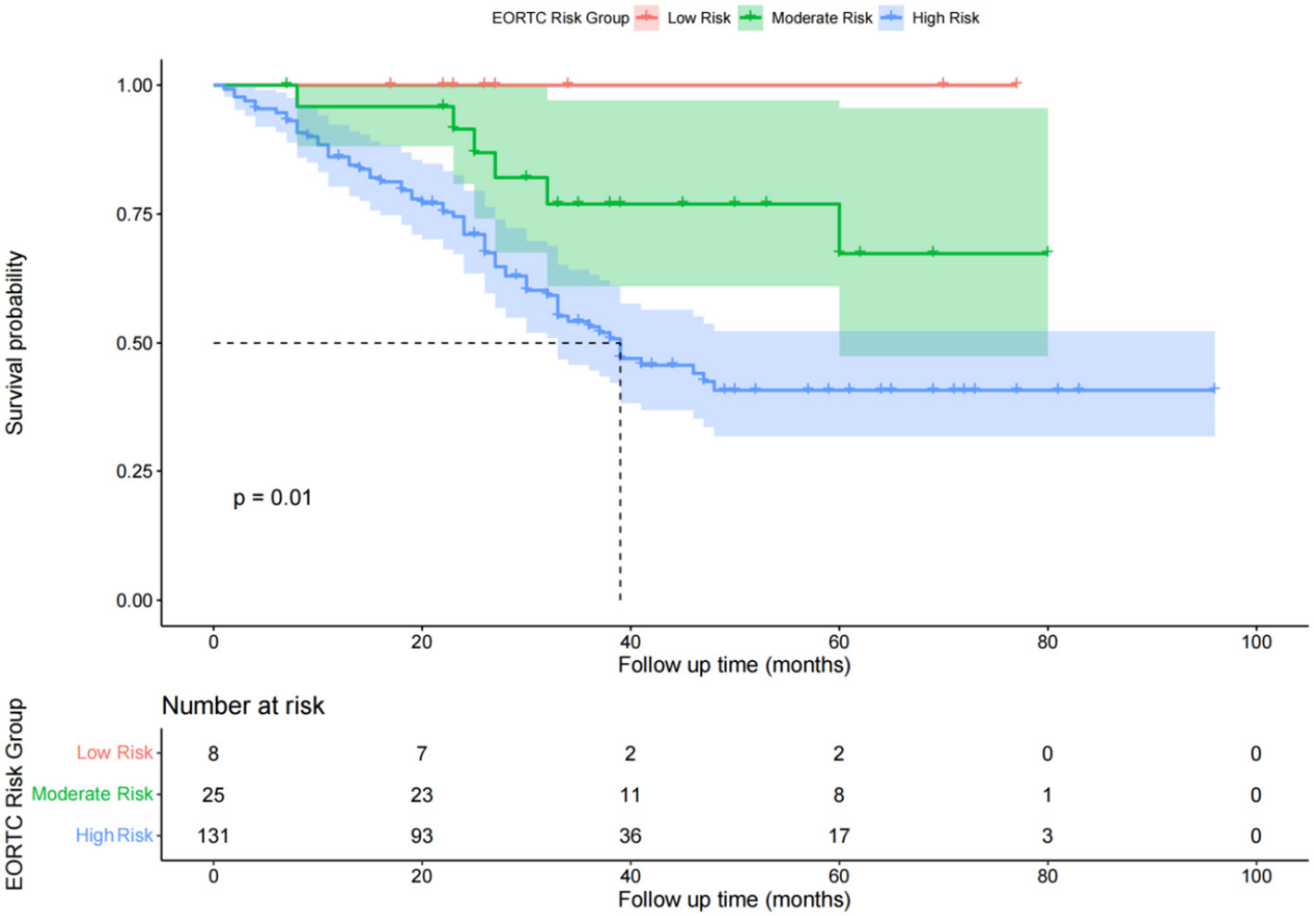
Diagram of postoperative overall survival function of patients in different risk groups of EORTC.

In the low-risk group, there were no cases of recurrence during the follow-up period. The RFS rates for the intermediate-risk group at 1, 2, and 3 years were 98.5%, 91.5%, and 76.9%, respectively. The cumulative RFS did not fall below 50% during the follow-up period, with an average RFS of 41 months and a standard deviation of 20 months for the intermediate-risk group. For the high-risk group, the RFS rates at 1, 2, and 3 years were 86.1%, 71.0%, and 43.1%, respectively. The median RFS was 38 months, and the average RFS was 34 months with a standard deviation of 20 months (**Figure 4**). The log-rank test showed a P-value of 0.03 (P < 0.05), indicating that the differences in RFS among the three groups were statistically significant, and that the lower the risk level, the better the survival prognosis.

### Univariate analysis of factors affecting RFS in NMIBC patients postoperatively

**Figure 5:**
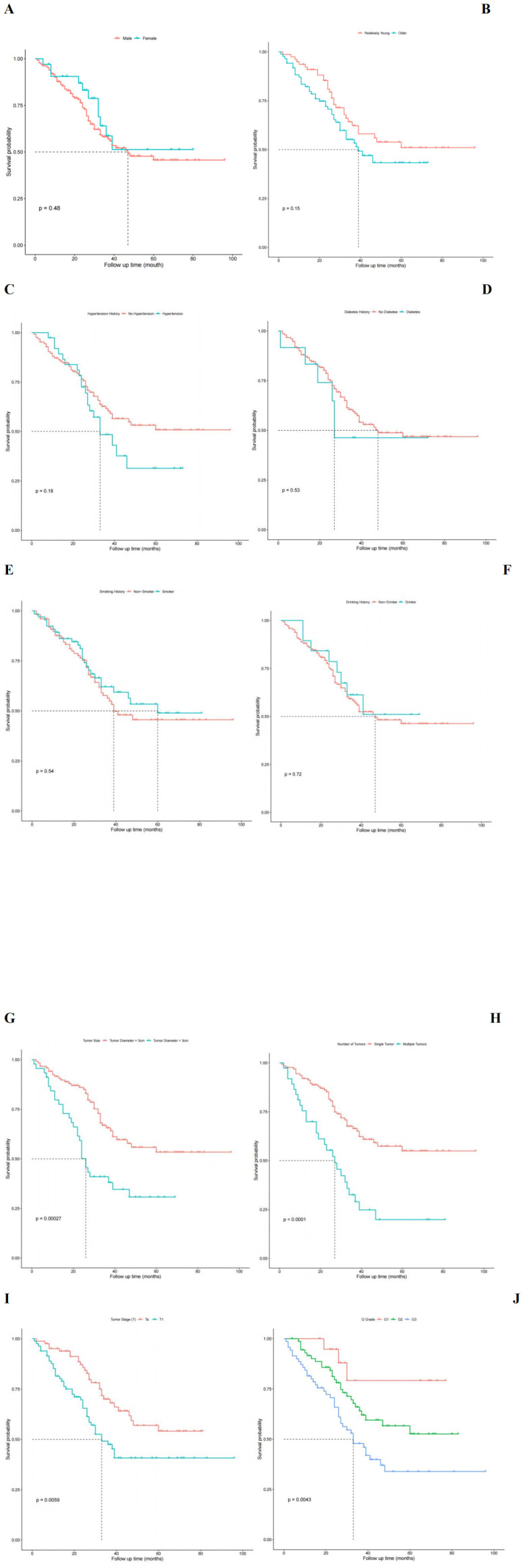
Univariate analysis influencing postoperative recurrence free survival in patients with NMIBC. **(A)** Survival functions for different sex groups. **(B)** Survival function for different age group. **(C)** Survival function plot with or without hypertension history. **(D)** Survival function plot with or without history of diabetes. **(E)** Survival function plot of smoking history. **(F)** Survival function plot of drinking history. **(G)** Survival function plot of tumor size. **(H)** Survival function plot of tumor number group. **(I)** Survival function of different T stages of tumor. **(J)** Survival function of different G grades of tumors.

Based on the patients’ RFS, we analyzed various factors that could potentially influence the prognosis of NMIBC using the Kaplan-Meier (KM) method. These factors included: (1) general information: categorized by gender, age, and other factors such as the presence or absence of hypertension history, diabetes history, smoking history, and the existence of alcohol abuse; (2) inflammatory response markers: P-CRP; (3) Tumor pathological characteristics: number of tumors, tumor size, T stage, and G grade. The differences in recurrence-free survival rates were compared using the log-rank test (**Figure 5**). The results showed that higher P-CRP values, the largest tumor diameter >3cm, higher T stage, higher G grade, and multiple tumors were independent risk factors affecting the RFS of NMIBC patients (P < 0.05).

### Multivariate analysis of factors affecting RFS in NMIBC patients postoperatively

Further multivariate analysis was performed using Cox regression to assess the impact of various factors on the postoperative RFS of NMIBC patients. The results indicated that five factors (P-CRP, T stage, number of tumors, tumor size, and G grade) had statistically significant effects on the postoperative RFS of NMIBC patients, even when considering potential confounding (P < 0.05). Specifically, the risk of tumor recurrence after TURBT for NMIBC patients in the high P-CRP group (>8.36) was 2.811 times higher than that for patients in the low P-CRP group (≤8.36) (95% CI: 0.204~0.625, P < 0.01). Additionally, pathological data such as the T stage (Ta or T1), tumor size (>3cm or ≤3cm), number of tumors (single or multiple), and G grade (G1, G2, or G3) all significantly influenced the RFS of NMIBC patients after TURBT (P < 0.05) (**Table 5**).

**Table 5:** COX regression analysis of multiple factors affecting relapse-free survival in NMIBC patients.

	B	SE	Wald	DF	F	Exp(B)	Cl of 95% Exp(B)	
							lower limit	upper limit
Variables								
pathological G2	0.479	0.619	0.599	1	0.439	1.615	0.480	5.433
pathological G3	1.212	0.608	3.981	1	0.046	3.361	1.022	11.054
P-CRP	1.033	0.286	13.015	1	<0.001	2.811	1.603	4.928
tumor size	0.837	0.260	10.371	1	0.001	2.309	1.388	3.844
T-stage	0.707	0.254	7.763	1	0.005	2.029	1.233	3.337
tumor number	0.992	0.272	13.324	1	<0.001	2.696	1.583	4.592

### Nomogram prediction model for recurrence in NMIBC patients

**Figure 6:**
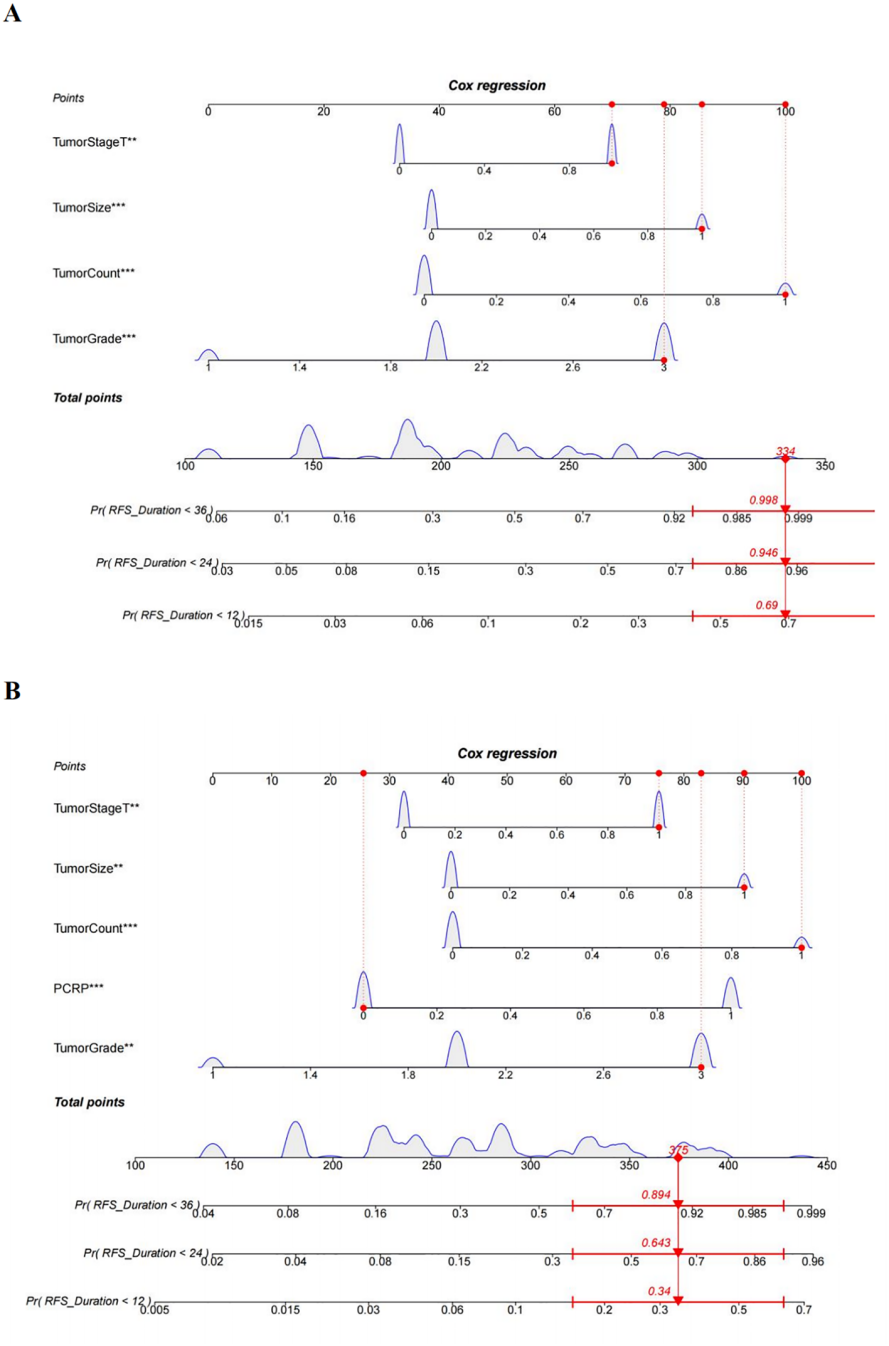
A nomogram diagram prediction model for patients with NMIBC recurrence. **(A)** Prediction model of postoperative recurrence-free survival in NMIBC patients without PCRP. **(B)** Prediction model of postoperative recurrence-free survival in NMIBC patients with PCRP. **means P<0.01, ***means P<0.001.

**Figure 7:**
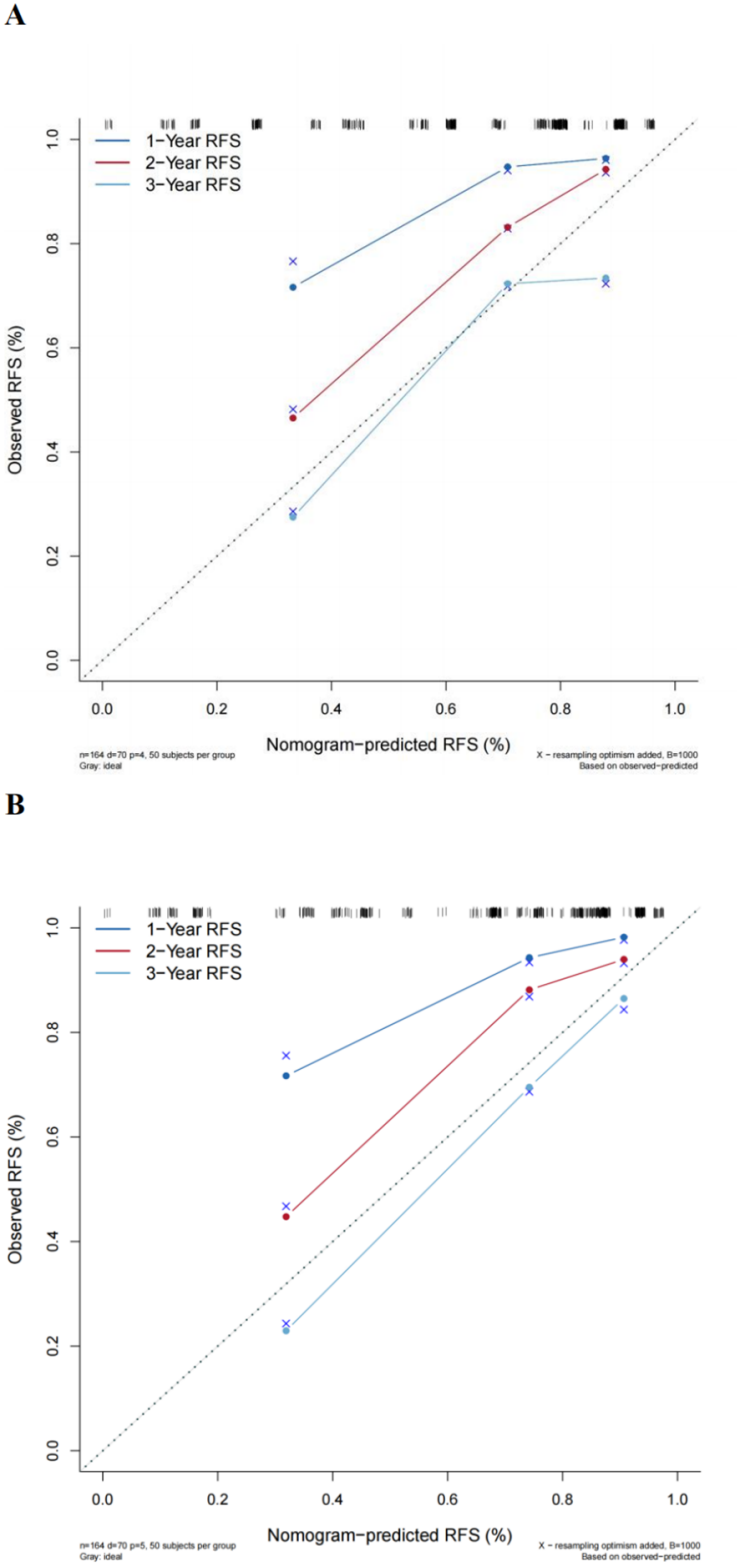
Comparison of the new model with the EORTC prognostic prediction model. **(A)** Traditional EORTC prognostic model. **(B)** New model calibration curves.

The multivariate Cox regression model indicated that preoperative P-CRP levels, number of tumors, tumor size, T stage, and pathological G grade have a significant impact on the recurrence-free survival (RFS) of NMIBC patients postoperatively. These influential factors can be incorporated into a Nomogram to estimate the median RFS and the risk of recurrence at 1, 2, and 3 years postoperatively, thereby constructing a prognostic prediction model for NMIBC (**Figure 6**). The concordance index (C-index) was 0.773, and by comparing the predicted values of the Nomogram with the actual outcomes using calibration curves, it was found that the deviations between the Nomogram predictions and the actual situations were within an acceptable range (**Figure 7**). This model can predict the prognosis based on the patient’s basic information. For example, for a NMIBC patient post TURBT with a TNM stage of T1, pathological grade of G2, a single tumor, and a tumor diameter >3cm, with a P-CRP value <8.36, the predicted RFS for this patient would be approximately 35 months, with a 1-year RFS rate of about 93.2%, 2-year RFS rate of about 81.8%, and 3-year RFS rate of about 65.5%.

### Comparison of the new model with the EORTC prognostic prediction model

A multivariate Cox regression model was constructed based on the variables included in the EORTC risk scoring system. The calculated concordance index (C-index) value was 0.742, which is significantly lower than the Nomogram model that included P-CRP (C-index = 0.773). Calibration curves also indicated that compared to the new model, the EORTC model had a greater deviation from the ideal predictive model (**Figure 7**). This suggests that the prognostic model incorporating P-CRP is more accurate in predicting the recurrence-free survival (RFS) of NMIBC than the traditional EORTC prognostic model.

## Discussion

In our investigation, we aimed to evaluate the prognostic significance of the preoperative platelet count and C-reactive protein (P-CRP) product in non-muscle invasive bladder cancer (NMIBC) patients and to develop a Nomogram to predict recurrence-free survival (RFS) post-transurethral resection of bladder tumors (TURBT). Our findings, derived from a retrospective analysis of 164 NMIBC patients, revealed that the preoperative P-CRP level, in conjunction with tumor number, size, T stage, and tumor pathological grading, were independent prognostic factors significantly associated with RFS. Notably, the Nomogram model that integrated P-CRP demonstrated superior predictive accuracy for RFS compared to the traditional model based solely on the European Organization for Research and Treatment of Cancer (EORTC) risk group parameters, with a concordance index (C-index) of 0.773, highlighting its potential utility in clinical practice for personalized prognostication.

A substantial body of literature has demonstrated that PLR (platelet-to-lymphocyte ratio), NLR (neutrophil-to-lymphocyte ratio), and others have a certain degree of influence on the prognosis of various malignant tumors (16–19). As a tumor closely associated with systemic inflammatory response, research on bladder cancer and its related inflammatory markers has been increasing annually (20–22). In this study, we included six blood inflammatory markers, including NLR, PLR, SII (systemic immune-inflammation index), PNI (prognostic nutritional index), AGR (albumin-globulin ratio), and P-CRP, to analyze their relationship with the RFS of NMIBC patients. We found that P-CRP had the best predictive ability for postoperative recurrence in NMIBC patients, and thus we chose P-CRP as our main research indicator.

P-CRP has been reported in recent years to influence the prognosis of various cancers. Hiroaki Saito et al. conducted a retrospective analysis of 453 patients with histologically diagnosed gastric adenocarcinoma who underwent curative surgery. They found that P-CRP was significantly associated with the depth of tumor invasion, tumor size, lymph node metastasis, disease stage, and patient age. Patients in the low P-CRP group had better prognosis (overall survival and disease-specific survival) than those in the high P-CRP group (P < 0.0001), and P-CRP was an independent prognostic factor for gastric adenocarcinoma (8). Masaki Morimoto et al., by analyzing data from 107 patients with pancreatic cancer who underwent pancreaticoduodenectomy or distal pancreatectomy, found that in terms of overall survival (OS), the prognosis for the high P-CRP group was worse than that for the low P-CRP group (P = 0.012) (7). However, it is worth noting that the cutoff value for P-CRP in Hiroaki Saito et al.’s study was 3.689, in Masaki Morimoto et al.’s study it was 1.782, and in this study, the cutoff value for P-CRP was 8.360. Although the cutoff values in these three studies were determined by the maximum point of the Youden index on the ROC curve, there are still differences in the specific values. The reasons for this may include several factors: First, each type of tumor has its unique biological markers and behaviors, which may lead to differences in the immune status of patients with different tumors; second, the race and genetics of different patients vary, and each study included patients with inherent differences, different types of surgery, and different postoperative living conditions, all of which can affect the cutoff value for P-CRP in the study; third, the number of patients included in each study is limited, which may also bias the selection of the optimal cutoff point.

In our study, the differences in white blood cell count, hemoglobin, neutrophil count, and monocyte count between different P-CRP level groups of NMIBC patients were statistically significant, and there were clear statistical differences in RFS between the different P-CRP groups, with the high P-CRP group having significantly lower RFS than the low P-CRP group. There could be several reasons for these findings: First, there is a certain correlation between platelets and tumor development. When tumor cells enter the bloodstream, they immediately activate platelets to form a microenvironment. Platelets recruit bone marrow cells through chemokines and bind with tumor cells on the vascular wall to form emboli, thereby protecting the tumor cells. Some growth factors derived from platelets help tumor cells acquire a mesenchymal phenotype and play a role in the development of capillary endothelium, promoting tumor activity (23–25). During the active process of the tumor, bleeding is inevitable, leading to differences in hemoglobin levels between groups. Second, CRP can act on tumors through many pathways and is a highly sensitive but relatively nonspecific marker of inflammatory response (26). Studies have shown that CRP levels are related to the number of neutrophils and lymphocytes (27, 28). There is also evidence that CRP is associated with the production of vascular endothelial growth factor, which plays an important role in tumor proliferation and invasion (29–31).

According to the EORTC prognostic grading standard, the cases included in this study were divided into low-risk, intermediate-risk, and high-risk groups. It was found that there were statistically significant differences in age composition between the different risk groups (P < 0.05), and the RFS of the low-risk group was higher than that of the intermediate-risk and high-risk groups. There were also statistically significant differences in RFS among patients from different groups (P < 0.05), which is consistent with the findings of previous scholars (32, 33). This also proves from another perspective that the patients we included can well represent the overall situation of NMIBC patients undergoing TURBT surgery. Among the different EORTC risk groups, there were statistically significant differences in three indicators: red blood cells, hemoglobin, and triglycerides (P < 0.05). The reason for the differences in red blood cells and hemoglobin may be due to the varying degrees of destruction of blood vessels around the tumor by bladder cancer in different risk groups, leading to differences in blood loss. The difference in triglyceride levels may be related to the varying impact of tumors with different pathological grades on the body’s lipid synthesis and uptake. Cancer cells can grow rapidly by enhancing the body’s ability to synthesize and uptake lipids (34).

In this study, we used the Kaplan-Meier method to perform univariate analysis on variables that might affect the RFS of NMIBC patients. Then, we included indicators with statistically significant differences from the univariate analysis into a multivariate Cox regression analysis. The results suggested that, among various factors interacting with each other, the preoperative P-CRP levels (>8.36, ≤8.36), number of tumors (single, multiple), tumor size (≤3cm, >3cm), tumor staging (PTa, PT1), and pathological grading (G1, G2, G3) of NMIBC patients were significantly associated with RFS (P < 0.05), and all were independent factors affecting the RFS after TURBT for NMIBC patients. The reasons for these findings may be related to the following points: First, pathological data such as the number of tumors, tumor size, and T staging have long been proven to be key factors affecting tumor prognosis. It has been well documented that BC has characteristics of heterogeneity. The higher the clinical staging and pathological grading, the more pronounced the atypia and heterogeneity of cancer cells, and the worse the prognosis for the tumor patient (5). Second, activated platelets participate in the regulation of tumor vascular stability and the tumor microenvironment, which can have an impact on tumor metastasis and invasion (35). CRP, on the other hand, contributes to tumor metastasis and invasion by promoting angiogenesis (36).

The Nomogram is currently a risk prediction model with high accuracy after surgery, which can help doctors devise more accurate individualized treatment plans and follow-up protocols. In this study, our model incorporated five different variables, each with varying degrees of relative risk. This allows us to quickly and succinctly evaluate and predict the prognosis of individual NMIBC patients post TURBT. The reason for using P-CRP and the Nomogram in this study is that platelets and CRP are generally measured routinely before surgery, eliminating the need for special tests, making it more cost-effective. Additionally, the Nomogram is simple and intuitive, with high practicality in clinical settings. Therefore, using this model to predict the prognosis of NMIBC patients is clinically feasible.

This study is an exploratory research on the use of preoperative P-CRP levels as a blood marker for predicting the recurrence-free survival (RFS) of NMIBC patients after TURBT, and we included pathological data (including the number of tumors, tumor size, T staging, and G grading) in our predictive model. Numerous studies have already proven that patients with BC associated with carcinoma *in situ* (CIS) have a poorer prognosis. Therefore, in this study, we excluded the variable of CIS, which enhanced the accuracy of our research findings. Compared to the traditional EORTC prognostic model, which is centered on patient pathological data, our model incorporated the blood inflammatory response marker P-CRP, and this model has a higher accuracy (C-index = 0.773 *vs*. C-index = 0.742), representing an advancement over the traditional risk stratification model for NMIBC postoperatively. Additionally, age and gender were not independent factors affecting RFS in NMIBC patients in this study, and thus, these factors were not included in the model. This discrepancy with other researchers’ studies may be due to the smaller number of patients included and the fact that they were all from a single center. It is worth mentioning that in this study, we exclusively used a domestic population as the follow-up sample and constructed a prognostic prediction model that includes the blood inflammatory response marker P-CRP. Through this model, we can clearly conduct individualized analysis for domestic NMIBC patients post TURBT and stratify their prognosis risks. This should provide a certain degree of assistance to clinicians in China for postoperative treatment and follow-up of NMIBC.

Our study’s results, which highlight the prognostic significance of preoperative P-CRP in NMIBC patients, contrast with the recent work of Caglayan A, Horsanali MO, who found no significant correlation between a broad panel of systemic immune response parameters and oncological outcomes in NMIBC (37). This discrepancy may be attributed to the different immune response markers examined, patient populations, or the specific analytical approaches employed. It is also plausible that the role of systemic immune response parameters in NMIBC prognosis is multifaceted and may vary depending on the tumor’s biological behavior and the host’s immune response.

While our study and others suggest certain inflammatory markers may predict outcomes in NMIBC, the absence of a consensus underscores the complexity of these relationships. For instance, studies have variously implicated neutrophil-to-lymphocyte ratio (NLR), platelet-to-lymphocyte ratio (PLR), and C-reactive protein (CRP) as potential prognostic indicators (18). However, as Caglayan A, Horsanali MO’s study indicates, not all investigations have found these markers to be predictive (37). This inconsistency may reflect the heterogeneity of NMIBC and the influence of confounding factors such as tumor stage, grade, and treatment modalities. Future studies with larger cohorts, standardized methodologies, and long-term follow-up are warranted to better understand these relationships and to determine the most reliable and clinically useful markers for predicting oncological outcomes in NMIBC.

Of course, our study still has some limitations. First, this study is retrospective in nature, which inherently has limitations in its design and ranks lower in the hierarchy of evidence-based medicine. Second, our data were sourced from a single center, and the clinical practices of the same center may have influenced the results to some extent, which would need to be supplemented by multi-center cohort studies. Third, the number of cases included in this study is not large, and the follow-up time is relatively short, with a total of 164 patients and a median follow-up time of only 30 months. More cases and longer, more comprehensive follow-up data are needed to refine the study. Fourth, the Nomogram model we constructed was only validated within the internal dataset and lacks sufficient persuasiveness. Therefore, our next goal is to use more external data for validation. Despite the many limitations of our study, we have still successfully demonstrated through statistical methods that P-CRP can serve as an independent prognostic factor for RFS in NMIBC patients postoperatively. We have also successfully constructed a Nomogram model that includes P-CRP and tumor pathological characteristics, which can be used to evaluate the probability of postoperative recurrence in NMIBC patients who have undergone TURBT and intravesical instillation therapy. This also contributes to the research on risk stratification in bladder cancer.

## Conclusions

The preoperative P-CRP level, tumor number (single or multiple), tumor size, tumor stage, and pathological grade of patients with non-muscle invasive bladder cancer (NMIBC) were significantly correlated with postoperative recurrence-free survival (RFS) (p < 0.05) and were identified as independent prognostic factors for RFS. The nomogram incorporating P-CRP demonstrated a notable predictive capability for RFS in NMIBC patients (C-index=0.773), classifying it as a moderately accurate model. Furthermore, the predictive performance of this model surpassed that of the model based on the EORTC risk grouping index (C-index=0.742).

## Data Availability

The original contributions presented in the study are included in the article/supplementary material. Further inquiries can be directed to the corresponding author.
